# Case definition terminology for paratuberculosis (Johne’s disease)

**DOI:** 10.1186/s12917-017-1254-6

**Published:** 2017-11-09

**Authors:** R. J. Whittington, D. J. Begg, K. de Silva, A. C. Purdie, N. K. Dhand, K. M. Plain

**Affiliations:** Sydney School of Veterinary Science and School of Life and Environmental Sciences, Faculty of Science, The University of Sydney, 425 Werombi Road, Camden, NSW 2570 Australia

## Abstract

Paratuberculosis (Johne’s disease) is an economically significant condition caused by *Mycobacterium avium* subsp. *paratuberculosis*. However, difficulties in diagnosis and classification of individual animals with the condition have hampered research and impeded efforts to halt its progressive spread in the global livestock industry. Descriptive terms applied to individual animals and herds such as exposed, infected, diseased, clinical, sub-clinical, infectious and resistant need to be defined so that they can be incorporated consistently into well-understood and reproducible case definitions. These allow for consistent classification of individuals in a population for the purposes of analysis based on accurate counts. The outputs might include the incidence of cases, frequency distributions of the number of cases by age class or more sophisticated analyses involving statistical comparisons of immune responses in vaccine development studies, or gene frequencies or expression data from cases and controls in genomic investigations. It is necessary to have agreed definitions in order to be able to make valid comparisons and meta-analyses of experiments conducted over time by a given researcher, in different laboratories, by different researchers, and in different countries. In this paper, terms are applied systematically in an hierarchical flow chart to enable classification of individual animals. We propose descriptive terms for different stages in the pathogenesis of paratuberculosis to enable their use in different types of studies and to enable an independent assessment of the extent to which accepted definitions for stages of disease have been applied consistently in any given study. This will assist in the general interpretation of data between studies, and will facilitate future meta-analyses.

## Background

Paratuberculosis (Johne’s disease) is caused by *Mycobacterium avium* subsp. *paratuberculosis (MAP)*. The organism commonly infects ruminants where it resides in macrophages in the small intestinal lamina propria and associated lymph nodes, triggering granulomatous inflammation and an enteropathy that is eventually fatal in many cases. The organism is not host specific, and infections have been reported from species as diverse as rabbits, cats and humans [1–3]. The economic losses in farmed livestock are due to lost milk production, weight loss and mortality [4–8]. In addition there are significant animal welfare considerations associated with this chronic wasting disease [9]. For these reasons, and also whether stated overtly or not the potential for *MAP* to appear in the human food chain, has stimulated the development of disease control programs for paratuberculosis in farmed livestock. In the face of limited resources and many unequivocal public health threats, these control programs satisfy public health agencies who recognize a link but not a causative relationship between *MAP* and Crohn’s disease [10], while topic specialists encourage the livestock industry to acknowledge the potential public health issue [11]. The requirement to reduce the prevalence of paratuberculosis has triggered studies to improve laboratory tests, vaccines and to explore the potential for breeding programs for disease resistance in livestock. However, difficulties in diagnosis and in the classification of the disease in individual animals have hampered research and efforts to overcome the progressive spread of paratuberculosis in the global livestock industry.

In one of the largest studies to date, involving detailed examination of more than 1000 cows using a battery of tests, Vazquez et al. (2014) [12] confirmed that most *MAP* infections are subclinical, and that the microbiological and pathological features of paratuberculosis leading to disease transmission are not dissimilar to tuberculosis in humans. In both humans and livestock, infected individuals carry pathogenic mycobacteria silently into new geographic areas [13–15]. It is a feature of chronic mycobacterial diseases in both humans and livestock that our ability to accurately classify individual cases and controls enables meaningful experimental design and analyses in many types of studies; generally these are aimed at better disease diagnosis, control, treatment and prevention (for example see [16, 17]). Descriptive terms that are applied to individual animals and herds such as exposed, infected, diseased, clinical, sub-clinical, infectious, resilient and resistant need to be described objectively and unambiguously so that they can be incorporated consistently into well-understood and reproducible case definitions. Herd-level case definitions can be developed from those applied to individual animals but are not dealt with in this paper.

**Fig. 1:**
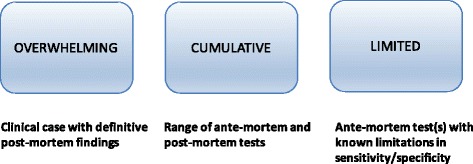
Conceptual ranking of evidence used to define a “case” in the paratuberculosis literature. Overwhelming evidence: indisputable diagnostic confirmation of clinical paratuberculosis. Cumulative evidence: range of ante-mortem and post-mortem tests applied and/or repeat testing at separate time points, combined with herd history, leading to greater certainty regarding true infection status. Limited evidence: use of ante-mortem tests such as the milk or serum ELISA and faecal culture, either alone or in combination; parallel interpretation of two or more tests (positive in any of the tests applied); uncertain diagnostic implications of combining information from more than one test

Case definitions allow for classification of individuals in a population for the purposes of analysis based on accurate counts. The outputs might include the incidence of cases (number of new cases per unit time and population at risk), frequency distributions of the number of cases by age class or more sophisticated analyses involving statistical comparisons of immune responses in vaccine development studies, or of gene frequencies or expression data from cases and controls in genomic investigations. Advances in technology and the availability of new testing platforms enable studies on immune, proteomic, genomic and metabolic disease signatures combined with sophisticated analytical methods, and these can be applied to animal models of paratuberculosis (for example [18]). These approaches aim to provide a greater understanding of disease pathogenesis and host susceptibility, however they ultimately depend on the classification of the status of individual animals. Hence there is a need to use consistent terminology and case definitions in order to establish the foundations for analysis. It is necessary to have agreed definitions in order to be able to make valid comparisons and meta-analyses of experiments conducted over time by a given researcher, in different laboratories, by different researchers, and in different countries. Indeed the lack of standard case definitions is recognized in tuberculosis research to hamper comparison of research findings, prevent best use of existing data and limit the management of disease [19]. This has led to promotion and comparison of standardized clinical case definitions in recent years, but it is already clear that case definitions conceived for one purpose, for example diagnosis of childhood tuberculous meningitis, may not be suitable for another, for example contact tracing, analogous to veterinary trace forward investigation (the problem here was lack of clinical signs in the latter being the first layer of classification in the former) [20, 21]. In this context the case definitions were intended to be used as standardized diagnostic criteria with a view to clinical intervention, and therefore the case definitions had properties of diagnostic tests (accuracy: sensitivity and specificity) [17]. For non-tuberculous mycobacterial infections in humans, adherence to American Thoracic Society diagnostic criteria to distinguish these from airway colonization was poorly associated with any difference in prognosis [22]. The issue is the use of consistent terms for analysis of diagnostic tests and then layering these to reach conclusions to enable classification of individuals. The same problem is evident in paratuberculosis, where animals are classified based on test outcomes; the tests can be used in series or in parallel, and so there will be variable accuracy of classification of the true infection or disease status. For example Zare et al. [23] classified animals as “test-positive” or “test-negative” based on ELISA alone or ELISA and faecal culture in parallel (parallel interpretation, individual is positive if either test is positive). The alternative would have been to require both tests to be positive (series interpretation, individual is positive if both tests are positive). Similarly Gonda et al. [24] used ELISA and faecal culture in parallel to classify animals in a herd as “infected” or non-infected for a genetic study. Collins et al. [25] used faecal culture results in parallel from tests repeated at up to three laboratories to classify animals as “a confirmed case of infection” to evaluate ELISA tests. Socket et al. defined subclinical infection to be isolation of *MAP* from faeces or organs of cows without clinical signs such as diarrhoea or weight loss [26]. Each of these definitions can result in a different pattern of grouping of individuals as cases or controls. Osterstock et al. (2010) [27] simulated faecal culture and ELISA results using reported sensitivity and specificity metrics, and demonstrated that the power to detect a genetic association using case definitions based on these tests was low. Conceptually, the evidence used to define a “case” can range from overwhelming to very limited when the accuracy of the aggregated testing procedure is considered (Fig. 1).

Paratuberculosis presents as a range of sub-clinical and clinical forms during the 1 to 14 year incubation period, and this chronicity of pathogenesis contributes to difficulties with disease characterization at each stage of progression [28]. The variation in disease presentation, the challenges of diagnostic test accuracy, when combined with the lack of consistency in application of terminology and case definitions has led to lack of agreement between studies, be they focused on validation of diagnostic tests, vaccine development or genome wide associations (for example see [29, 30]).

## Scope

In this paper, a brief discussion of the specific requirements for individual animal case definitions in studies related to diagnostic tests, genetics/genomics and vaccines is presented. Diagnostic tests for paratuberculosis, and their attributes are briefly introduced. This is followed by an explanation of terms that are commonly used in the context of paratuberculosis. These terms are then applied systematically in an hierarchical flow chart to enable classification of individual animals using available information such as herd history and objective diagnostic tests. Areas of uncertainty remain with certain animal classifications, and these are highlighted. We propose descriptive terms for different stages in the pathogenesis of paratuberculosis to enable their use in case definitions for different types of studies (diagnostic test evaluations, prevalence estimation, certification of disease freedom, vaccine efficacy and genomic studies). Case definitions can be precisely defined, should be linked to an understanding of pathogenesis, and should be consistent between studies. However, the stringency with which a case definition can be met will differ between studies according to resources and other practical considerations, and the accuracy of animal classification (i.e. the sensitivity and specificity) will also vary. For this reason it is important that guidance be provided to enable self-assessment or indeed an independent assessment of the extent to which case definitions are met in any given study.

## Contexts for application of specific terms and case definitions

### Diagnostic test evaluation

**Fig. 2:**
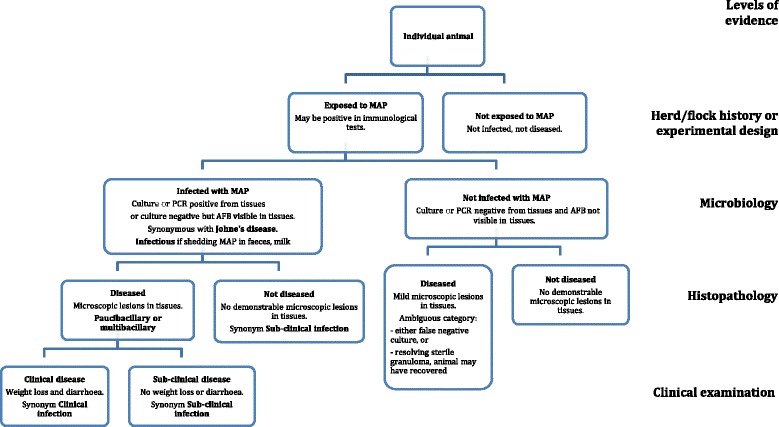
Primary dichotomous classification of animals exposed to *MAP* using a systematic and structured diagnostic approach. AFB, acid fast bacilli

One of the most important applications of case definition is in diagnostic test evaluation. The World Organisation for Animal Health (OIE) recommends that diagnostic tests be evaluated after explicitly stating the purpose of the test (to ensure that a test is fit for purpose, for example “to detect animals that can transmit the disease”) and more generally “to accurately predict the infection or exposure status of the animal or population of animals” [31]. In 2008 Nielsen and Toft reviewed the accuracies of diagnostic tests for paratuberculosis and made distinctions between non-mutually exclusive categories of infected, infectious and affected animals, which they termed “target conditions” [30]. Their epidemiological approach, which recognized the long latent phase of the infection (pathogenesis), enabled test accuracy to be summarized based on stratification of the stages of the disease. This approach is well-recognized, for example in an assessment of direct faecal PCR test accuracy based on stratification of animals in the population according to the severity of their histopathological lesions [32]. Nielsen and Toft (2008) [30] recommended that diagnostic test evaluations be more stringent in future, including consistent classification of subjects and avoidance of variable case definitions. In their conceptualization, the target condition is linked to the purpose of a diagnostic test and to pathogenesis, while the case definition is “a practical description of the target condition” [33]. In other words, the case definition is chosen to suit the needs of the individual study, often involving pragmatic compromise. However, the case definition should be constrained by rules to reflect the target condition [33]. The problem with this approach is that case definition is flexible, and studies conducted for different purposes are not easily comparable because there is lack of consistency in the use of terms that underpin the various possible case definitions. An alternative view is that the target condition is a general statement related to the purpose of a diagnostic test; note that there is little difference between purpose and target condition, as shown in Figure 2 in the article by Nielsen et al. 2011 [33], while case definition is a more precise statement, or an algorithm for diagnostic test inclusion and interpretation. This is also how case definitions have usually been applied in the tuberculosis literature (for example [17]).

### Genetics/genomics/genome wide association studies

The purpose of a genome wide association study (GWAS) is to find a relationship between defined genes/alleles and disease characteristics (i.e. a phenotype) at population level, with a view to understanding the relationship between genotype and phenotype at individual animal level. If there is a strong relationship, it may be possible to select genetically resistant individuals for breeding, or alternatively cull susceptible animals to exclude them from the breeding population. Clearly in this context an accurate classification of phenotype is important, and this can be enabled by appropriate and consistent use of case definitions. The lack of consistency of phenotypic classification of animals is one important reason for lack of agreement in the results of different GWAS studies for paratuberculosis [29, 34, 35]. Mostly the discrepancies are due to application of different diagnostic tests with different sensitivities and specificities, at different times in the pathogenesis of the disease, resulting in potential misclassification bias of both cases and controls [34]. Approaches to avoid misclassification bias, given that the genome sequence of an individual is fixed, include repeated testing over time to cover changing disease state in the animals [36], application of multiple ante-mortem diagnostic tests and interpretation of test results in parallel [37, 38] and post-mortem examination which enables culture of tissues and histopathological examination [36, 39]. These steps would increase diagnostic sensitivity, which is often lacking in paratuberculosis studies. However, there may be circumstances where series interpretation of test results is useful to avoid misclassification bias, particularly where specificity of a screening test is suboptimal, for example interferon-*γ* tests for paratuberculosis in calves [40]. While principles of phenotypic classification are well understood there is as yet no standard upon which to guide genetic studies for paratuberculosis.

### Vaccine development and evaluation

Research and development on new vaccines to protect against paratuberculosis will continue [41] until an improved formulation is developed that has better efficacy and safety than those currently available [42–44]. The efficacy of vaccines for paratuberculosis can be measured by reductions in viable *MAP* in tissues, histopathological lesions, faecal shedding of *MAP*, incidence of clinical disease or production of sterile immunity [45, 46]. Researchers should consider using all of these measures as part of a defined animal outcome to determine candidate vaccine efficacy. To avoid misclassification bias of post vaccination outcomes, accurate case definitions using well understood diagnostic measures should be used [30].

The outcome of infection in a vaccinated ruminant could include recovery if vaccination occurs post exposure to *MAP*. Subharat et al. (2012) [47] observed a reduced severity of infection in cattle from 7 to 15 months post vaccination, while Dennis et al. (2011) [48] observed recovery in naturally infected sheep. Description of the spectrum of disease within the unvaccinated control population is also important to accurately measure vaccine efficacy; if the onset of clinical disease in control animals is unnaturally early or prevalent for that species, the protective response of the vaccine may be overwhelmed [49]. Conversely, if none of the animals from the unvaccinated control group develop clinical disease it is hard to establish whether the vaccine is protective against this important outcome, even if histological lesions and *MAP* recovery is recorded. If validated, defined early markers/profiles of infection outcome in the natural host [50] could be used in vaccine efficacy screening instead of conducting screening studies in cell cultures or mice [51].

### Disease diagnosis and regulatory action

Paratuberculosis is a notifiable disease in some countries, and accurate diagnosis is important prior to application of control measures which may require stamping out, culling or quarantine measures. Establishing the prevalence of paratuberculosis in a region, or assurance that the infection is absent from a region are also important activities. Trace forward investigations, trace back investigations and confirmation of true infection status following positive results in screening tests are common scenarios. There is some guidance from the OIE on test procedures [52] and also in individual countries (for example Australia [53]) but in general there is no agreed terminology for so-called “target conditions”.

## Characteristics of diagnostic tests for paratuberculosis

Many of the terms and case definitions for paratuberculosis depend on the results of objective diagnostic tests and historically these have been widely applied to classify animals with paratuberculosis. There are numerous protocols for conducting tests for paratuberculosis (for example see OIE [52]). In general they can be classified as tests for the pathogen (culture or direct PCR of faeces, tissues or milk), or tests for the host’s immune response (antibody detection ELISA on serum or milk, various assays for cell mediated immunity such as delayed type hypersensitivity tests) or tissue inflammatory response (gross pathology and histopathology). Test accuracy can be described in terms of sensitivity (the proportion of sick animals that are detected) and specificity (the proportion of healthy animals that test negative), but all are imperfect, i.e. false positive and false negative test outcomes occur [30]. An indication of the accuracy of various tests is provided in Table 1. The lack of perfect tests creates problems in diagnostic test evaluations as newer tests such qPCR are often compared to less sensitive/specific established tests, resulting in apparent lower sensitivity and specificity estimates for newer tests. This has led to an increase in the use of Bayesian latent class models for diagnostic test evaluations, and recommended standards for these [54, 55].

**Table 1:** Temporal applicability and accuracy of diagnostic tests for paratuberculosis in sheep and cattle

Test	Stage of disease when positive	Potential sensitivity^a^	Potential specificity^b^
Serum ELISA	Mid, late	Low to high^d^	Moderate to high
Delated type hypersensitivity	Early, mid	Moderate to high	Moderate
Interferon gamma assay	Early, mid	Unknown	Unknown
Faecal smear	Mid, late	Low^e^	Low to moderate
Faecal culture	Early^c^, mid, late	Low to high^d^	High
Faecal qPCR	Early^c^, mid, late	Moderate to high^f^	High
Tissue culture	Early, mid, late	High	High
Gross pathology	Late	Low to moderate	Low to moderate
Histopathology	Mid, late	Moderate to high	High
Clinical signs	Late	Low to moderate	Low to moderate

Adapted from Whittington and Sergeant (2001) [28] and Nielsen and Toft (2008) [30]. The terms low, moderate and high indicate ranges for sensitivity of <40%, 40–70% and >70%, respectively; corresponding values for specificity are <80%, 80–95% and >95%, respectively

^a^Proportion of truly infected/diseased animals that test positive

^b^Proportion of truly non-infected/healthy animals that test negative

^c^Transient, for a few months commencing a few months after infection

^d^High sensitivity possible in late stage of disease

^e^Due to low analytical sensitivity

^f^Based on sensitivity similar to culture in liquid medium

Mismatches between the results of different tests for paratuberculosis are common: tissue culture results may not agree with histopathology; faecal culture may not agree with antibody ELISA. For example, *MAP* was cultured from the tissues of about 30% of sheep and 7% of cattle that did not have histopathological lesions [12, 56]. More recently, in a series of trials conducted at the University of Sydney between 2006 and 2012 involving over 400 sheep that were intensively monitored and received a full necropsy, 13% of the sheep positive for *MAP* by tissue culture had no detectable histopathological lesions across six different regions of the gut and associated lymph nodes (unpublished data).These discrepancies can be readily understood in terms of stage of pathogenesis, site of sample selection and amount of sample examined, and biological/physical properties of the tests (for discussion see [28]). These factors mean that for accurate classification of animals it is often necessary to apply a range of tests, and to do this at more than one time point so as to capitalize on stages of pathogenesis that favour one type of test over another. At any particular time, the stage of pathogenesis in any given individual may be unknown.

### Culture of MAP

This test is usually considered to have 100% specificity [30, 57] assuming the appropriate confirmatory tests are used. However, the sensitivity is imperfect because of stage of disease, sample selection and sample decontamination [28, 58]. Sensitivity estimates for culture of faeces range from 16 to 74% across species and stages of disease [30]. Culture of intestinal tissues is the most sensitive way to confirm infection at individual animal level. In addition, culture results can be quantitative [59, 60] and culture performed in liquid medium is more sensitive than culture on solid media (reviewed in [57]). Due to intermittent shedding of *MAP* in faeces, the more frequently samples are collected and tested the greater the number of shedding individuals that can be detected [61].

### Direct faecal PCR tests

Direct faecal PCR tests have been developed in recent years [32, 62–65]. If results are normalized against a DNA standard curve, such tests provide quantitative results which correlate with stage of disease [65]. High *MAP* DNA quantities are more likely to represent true infection rather than passive (pass through) shedding of the bacterium [66]. There are few validation studies of test accuracy for direct faecal PCR tests, but sensitivity and specificity of some tests may be similar to faecal culture [65].

### Anti-MAP antibody ELISA

A positive result in antibody ELISA is defined by the test kit manufacturer, or the user in terms of sample-to-positive (S/P) or another normalized value. The results of many studies have shown that the specificity of the assay is usually high [30] except in certain geographical locations where specificity may be low due to exposure to environmental mycobacteria [67]. Antibody ELISA on serum samples or milk samples has relatively low sensitivity [30, 68] except in late stages of the disease. Positive results can indicate exposure to *MAP* [69]*.*

### Interferon-gamma (IFN-γ)

A positive result is defined in terms of S/P or another normalized value. There have been few validation studies [30] and the specificity of the assay may vary depending on the species, age of the cohort tested and the experimental protocol used. Positive results have been associated with exposure to *MAP* but not necessarily with infection [50, 70]. The assay offers the potential to detect exposure to *MAP* early in the life of an animal, however the sensitivity of the IFN- *γ* assay is unknown [71].

### Lymphocyte proliferation assay

This is a research tool. A positive result is defined by the laboratory; there have been no validation studies but positive results have been associated with exposure to *MAP* [16, 72–74].

### Necropsy

This enables a thorough examination of relevant tissues (particularly those of the ileum and associated lymph nodes) and their collection for *MAP* culture as well as histopathological examination. The greater the number of sites in the intestine examined the more likely it is that infection and or disease will be detected [75].

### Gross pathology

The gross pathology associated with *MAP* infection, which includes enlargement of mesenteric and ileocaecal lymph nodes and thickening of the intestinal mucosa is not specific to paratuberculosis, and gross changes do not occur in all affected animals [76–80]. Cording of lymphatic vessels in the serosa, mesentery and associated lymph nodes is due to advanced granulomatous inflammation, usually in late stages of pathogenesis.

### Histopathology

Objective lesion scoring criteria have been published and are widely used in research applications [12, 48, 81–84]. These systems have multiple categories to describe the extent, severity and nature of the granulomatous lesions that characterize paratuberculosis. The Perez classification [81], like others is categorical and not necessarily ordinal. However, it is likely that there is an order of progression of lesions from mild to severe, and from paucibacillary to multibacillary, based on the results of sequential biopsies [48]. Using the system of Perez et al. (1996) [81] this is from 1 (mild focal) to 2 (focal) to 3a (multifocal), and then to either 3b (multifocal to diffuse, multibacillary) or 3c (multifocal to diffuse, paucibacillary). It is important that relevant tissues are examined. In general, the terminal ileum and ileocaceal valve region and nearby lymph nodes are considered to be predilection sites, but lesions extend more widely along the intestine as the disease progresses [82, 85, 86]; examination of a wider range of intestinal sites and associated lymph nodes is recommended to be more confident that lesions are not present.

## Definitions of terms

The following terms are based on the systematic collection of objective historical evidence, clinical signs and laboratory test results that can be organized hierarchically into a logical framework for conceptualization of case definitions. The hierarchy is presented in Fig. 2.

### Paratuberculosis or Johne’s disease

This is a comprehensive term to describe all forms of infection with and disease caused by *MAP*. It does not necessarily imply that the animal has outward signs of disease.

### Exposed

The animal has been exposed to *MAP*, either through direct or indirect contact with known-infected animals, pasture or bedding or milk, or through an experimental infection. This classification is based on history, a field epidemiological assessment, or direct knowledge of the experimental design. It might be possible to determine whether there was exposure to a potentially infectious dose, or alternatively whether the exposure was unlikely to be sufficient to lead to infection.

### Infected

The animal is infected with *MAP* based on a microbiological assessment. Infection is defined by culturing *MAP* from the tissues of the animal, or by demonstrating *MAP* in the tissues by PCR. This is a definitive test process that is conducted post mortem or through biopsy. Culture of faeces can provide indirect evidence of infection. The presence of *MAP* in faeces can be due to pass through (passive shedding) when the animal is ingesting *MAP* from the environment, and although this provides very strong evidence of exposure, it is not confirmation of infection. Pass through occurs for 7–10 days after a single oral exposure in small ruminants and cattle [40, 87–93]. Detection of *MAP* in faeces on more than one occasion (i.e. from independent samples collected on different days) provides more information and is suggestive of infection. In one study, 80% of cows with ≥1 faecal sample positive in culture also had a positive culture test result from their intestinal tissues, confirming true infection [94]. In a 2.5 year trial, 80% of *MAP* exposed sheep grazing together on pasture were infectious (≥ 1 positive fecal culture) but only 45% were infected [95]. Faecal shedding in the uninfected sheep halted 6 months after exposure. Except in special circumstances (see below), microscopic examination alone of tissues collected at post mortem or biopsy is not sufficient to define infection because acid fast bacilli (AFB) other than *MAP* may be present in tissues [80]. Also except in special circumstances, positive ELISA test results alone are not sufficient to define infection because false positive immunological reactions are possible in animals that have been exposed to environmental mycobacteria; this can occur sporadically or because of geographic location [67]. Due to the potential for pass through of *MAP*, and for spurious ELISA results, positive faecal culture and blood or milk antibody ELISA test outcomes on more than one sampling occasion increase the confidence about infection of the animal, but are not definitive.

#### Clinically infected

The animal is infected and has clinical signs (see Clinical Disease below).

#### Subclinically infected

The animal is infected but does not have clinical signs (see Subclinical Disease below).

### Infectious

The animal is shedding viable *MAP* in its faeces or milk. This classification is based on a microbiological assessment and is defined by positive results in faecal or milk culture. The analytical sensitivity of faecal culture is ≥100 bacilli per gram of faeces [57]. As the infectious dose is not accurately known [49, 96], any detection of *MAP* in faeces by culture indicates that the animal is potentially infectious. The amount of shedding and therefore the relative degree of infectiousness can be determined by culture, and animals can be graded into light, medium or heavy shedders or super-shedders [97, 98]. In the case of pass through shedding, the animal may be considered to be infectious at the time it was shedding. In special circumstances (see below) such as experimental inoculation trials where live bacteria have been administered orally and infection is likely, or where prior faecal culture tests were positive, shedding may be defined by faecal PCR or faecal smear stained with Ziehl Neelsen (ZN), acknowledging that these tests do not distinguish between live and dead bacteria. Quantitative faecal PCR can provide information on the level of shedding [65].

### Diseased

The animal has demonstrable histopathological lesions consistent with *MAP* infection. It may also have gross pathological lesions in the intestinal tissues and associated lymph nodes. Most but not all diseased animals will also have demonstrable infection (Fig. 2). Gross lesions are not specific for paratuberculosis and may be absent [76]. There is an ambiguous category where exposure occurred, microscopic lesions are present but the organisms cannot be demonstrated; this category includes recovered cases.

#### Histopathology positive

Granulomatous lesions attributable to *MAP* are present. The morphological diagnosis can be refined using one of the lesion classification schemes [12, 48, 81–84] to assess the stage of disease and infer the degree of infectiousness, because multibacillary lesions are correlated with heavy faecal shedding [50]. Specific terminology is used in Australia for diagnostic purposes [53]: “a diagnosis of ‘lesions consistent with *MAP* infection’ is indicated if in any one section, one or more single giant cells and/or one or more accumulations of epithelioid macrophages are observed in the intestinal lamina propria and/or lymph node cortex with the presence of at least one acid fast organism (AFO) morphologically consistent with *MAP”*; a finding ‘suggestive of *MAP* infection’ is indicated if AFO are not observed. Arguably, microscopic evidence is incomplete unless AFB in the lesions are confirmed to be *MAP*, for example by PCR, or are cultured from the tissues, because other species of mycobacteria can induce similar lesions. Lesion progression is not well described and the distinct categories defined below represent a continuum; for example early lesions in the category of 3a [81] may be paucibacillary or multibacillary precursors.

#### Paucibacillary lesion

These histopathological lesions do not contain large numbers of AFB [81, 82]. In the classification of Perez et al. (1996) [81] these are typically score 3c lesions, which are quite widespread, but the more focal lesion grades of 1, 2, and 3a usually contain no or few detectable AFB.

#### Multibacillary lesion

These histopathological lesions are typically widespread or diffuse and contain large numbers of AFB [81, 82]. In the system of Perez et al. (1996) [81] these are termed 3b lesions, but some of the earlier lesions in the 3a category can also contain large numbers of AFB.

#### Clinical disease

The animal has disease and associated clinical signs, specifically demonstrable weight loss, measured as ≥10% body weight loss over one month [99], and/or is in low body condition score relative to the majority of animals in the flock/herd. Objective visual body condition scoring systems are used internationally [100]. Cattle may have diarrhoea; usually this is profuse and watery. Small ruminants usually do not have watery diarrhoea, and often have normal faecal pellets.

#### Sub-clinical disease

The animal is diseased but does not have clinical signs attributable to paratuberculosis.

The clinical signs of paratuberculosis are not specific. Weight loss and diarrhoea may occur for a variety of reasons, therefore clinical signs alone cannot be used to define paratuberculosis. Where clinical signs are observed, they can be ascribed to paratuberculosis only when microscopic pathology in the intestine is observed that is consistent with paratuberculosis, with or without gross pathology. Except in special circumstances, culture of *MAP* from tissues or faeces cannot be used as the only test to confirm that clinical signs are due to *MAP* because clinical signs are not due to infection per se; this is clear because animals with paucibacillary or multibacillary lesions can succumb to clinical disease; these are cases with widespread severe lesions rather than mild lesions [85].

### Resistant/resilient

**Fig. 3:**
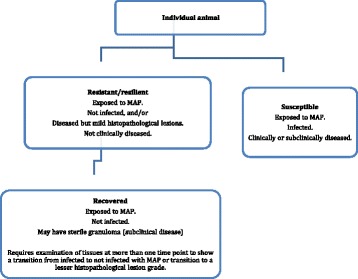
Secondary classification of animals exposed to *MAP* based on their susceptibility or resistance to infection and disease, defined using the diagnostic approach in Fig. 2. Recovered is a subgroup of resistant/resilient, defined by more stringent evidence

It may be possible to classify animals with resistant phenotypes in some circumstances (Fig. 3). Conceptually, resistant/resilient animals are known to have received an infectious dose of *MAP* at an age when they were susceptible but the infection does not establish, does not progress, or remains in a dormant state so that when the animal is examined at necropsy, the infection cannot be detected by culture of tissues and there is no evidence of disease in the histopathological examination.

### Recovered from paratuberculosis

The concept of recovery from paratuberculosis depends on strong evidence in order to be accepted. It is a subset of resistant/resilient (Fig. 3). Recovery from paratuberculosis has seldom been demonstrated; it means the elimination of a demonstrable infection [48]. Proof of recovery requires detailed examinations at more than one time point: the animal is shown to be infected and may be diseased at the first time point and is not infected at a subsequent time point. Recovered animals may have residual histopathological lesions from which *MAP* cannot be cultured (sterile granuloma), i.e. have evidence of disease, but the lesions should be mild or of a lower grade than those observed at an earlier time point.

To show resistant/resilient and recovered, exposure to a known infectious dose of *MAP* can be inferred in two ways. Firstly, using a recognized experimental infection model in which the animal was exposed to *MAP* at a susceptible age by direct inoculation on more than one occasion [49, 99, 101]. Susceptibility of cattle and sheep to MAP infection is greatest before about 6 months of age [102, 103]. A second way is in natural settings where paratuberculosis has been observed. In both contexts, a spectrum of disease is subsequently observed in the cohort of animals, showing that the exposure led to infection and that the host/environmental conditions were conducive to disease expression. Laboratory tests can be used to confirm exposure of individuals, for example a positive result in the IFN-γ test, lymphocyte proliferation assay, anti-*MAP* antibody ELISA and/or faecal culture [30, 50, 69, 70, 72, 99, 104–106] can indicate exposure. *MAP* DNA quantities in faeces assessed by direct quantitative faecal PCR test must be low, that is, there is no evidence of heavy shedding of *MAP* [32, 65]. It is well known that young animals shed *MAP* for a short time commencing several months post exposure; then, after a potentially long latent period, active shedding may recommence as the disease progresses [95, 107, 108]. Therefore, to confirm resistance it is necessary to show that if faecal shedding occurred in a young animal, it ceased, and then there was a lengthy period during which the animal did not shed *MAP*; consequently this assessment can be made only in adult animals after several years. Following necropsy, *MAP* cultures on tissues collected from multiple sites in the gut including the ileocaecal valve and associated lymph nodes must be negative and histopathological lesions if detected must be mild (for example Perez score < 2 [81]). Necropsy examination must be conducted after the animal has had enough time to develop identifiable histopathological lesions, i.e. in adults preferably after several years. Further research is required to properly define these intervals for verification, but the scale is likely to be measured in years.

### Susceptible

Susceptible animals are those that develop infection and disease after natural or experimental exposure to *MAP* (Fig. 3).

### Special circumstances

Where objective information is available to increase the level of confidence that *MAP* is involved, variation in the criteria above that define infected, infectious and diseased are possible, and it is not necessary to prove that mycobacteria are *MAP*. This is in the context of experimental infection when an animal has been inoculated with or deliberately exposed to *MAP*, a spectrum of disease has been demonstrated after exposure in the cohort, and mycobacterial agents such as *M. bovis* are not present in the population. Natural exposure to *MAP* may be certain, specifically when herd history and expert opinion exists to determine that an animal has been exposed, for example in a herd or flock pasture grazing scenario where there is a high prevalence of infection, or where dairy calves have been exposed to known-infected dams and other mycobacterial diseases are believed not to be present in the population. In these circumstances, AFB observed in tissues or faeces are assumed to be *MAP*; characteristic histopathological lesions without AFB are assumed to be due to *MAP*; anti-*MAP* antibody ELISA positive tests on more than one sampling occasion are assumed to be due to *MAP* exposure; an animal is assumed to be infectious where a prior faecal culture test was positive and later there are positive faecal smear or PCR results (acknowledging that faecal smear and PCR do not distinguish between live and dead bacteria).

## Incorporation of terms in case definitions

To be useful, case definitions should be written using terms that are well understood; the descriptions provided above are designed to this end. In addition, where the results of diagnostic tests are used to assign individuals to categories, these tests must be described, and their accuracy documented. This enables an assessment of the extent to which particular case definitions are based on sound data, are consistent and are likely to be “correct”. For example if the only evidence of paratuberculosis is an ELISA test result, the level of confidence that an animal is infectious is very low, and if the ELISA test result is negative, it should not be assumed that the animal is not infected.

## Conclusions

In this paper, we have proposed descriptive terms for inclusion in case definitions for different stages in the pathogenesis of paratuberculosis. This evolved while planning large scale trials and from our perception that there was a need more broadly for discussion leading to consensus regarding the inclusion of information and reporting of case definitions. Our aim is to propose a framework for animal classification and promote further research. The terminology provided here will be useful in diagnostic test evaluations, prevalence studies, certification of disease freedom, studies of vaccine efficacy, genome wide association studies and also in routine diagnosis of paratuberculosis. Considerations such as time, budget and practicality often determine study designs, however, it should be possible to make an independent assessment of the rigor and extent to which case definitions are met in any given study by determining whether definitions for stages of disease have been applied consistently. This will assist in the general interpretation of data between studies, and will facilitate future meta-analyses.

## Abbreviations

AFB: Acid fast bacilli
AFO: Acid fast organism
DNA: Deoxyribonucleic acid
ELISA: Enzyme-linked immunosorbent assay
GWAS: Genome wide association study
IFN-γ: Interferon-gamma
MAP: *Mycobacterium avium* subsp. *paratuberculosis*
OIE: Office International des Epizooties, World Organisation for Animal Health
PCR: Polymerase chain reaction
qPCR: Quantitative real time PCR
ZN: Ziehl Neelsen
